# Direct binding of Magi2 to the USH1G protein SANS links the periciliary USH protein network to endocytosis

**DOI:** 10.1186/2046-2530-1-S1-P14

**Published:** 2012-11-16

**Authors:** K Bauss, A Klinger, V Etz, T Maerker, E van Wijk, Rachel Roepman, H Kremer, U Wolfrum

**Affiliations:** 1 Cell and Matrix Biology, Inst. of Zoology, Johannes Gutenberg University of Mainz, Germany; 2 Dept of Human Genetics, Nijmegen Centre for Molecular Life Sciences, Nijmegen, Netherlands; 3 Institute for Genetic and Metabolic Disease, Nijmegen Centre for Molecular Life Sciences, Nijmegen, Netherlands; 4 Dept of Otorhinolaryngology, Head and Neck Surgery, Nijmegen Centre for Molecular Life Sciences, Nijmegen, Netherlands; 5 Nijmegen Centre for Molecular Life Sciences, Nijmegen, Netherlands; 6 Donders Institute for Brain, Cognition and Behaviour, Radboud University Nijmegen Medical Centre, Nijmegen, Netherlands

## 

The human Usher syndrome (USH) is the most common form of combined deaf-blindness. The encoded molecules are integrated into protein networks by scaffolds including the USH1G protein SANS (scaffold protein containing ankyrin repeats and SAM domain). Previous studies indicated SANS´ participation in vesicle transport and cargo handover at the periciliary region of photoreceptor cells. To decipher the precise cellular role of SANS, we searched for interacting partners. Therefore we adopted a yeast-2-hybrid screen of a retinal cDNA library using SANS´ C-terminus as bait. Amongst others we identified the MAGUK protein Magi2 (membrane-associated guanylate kinase inverted-2) as putative binding partner. Magi2 is known as scaffold protein involved in endocytosis at synapses. We confirmed Magi2 as direct interaction partner of SANS by complementary interaction assays. In addition, correlative light and electron microscopy showed partial co-localization of SANS and Magi2 in the periciliary region of photoreceptor cells. Furthermore, immunoelectron microscopy revealed the association of Magi2-SANS complex with membranous vesicles in this region. These vesicles may represent either vesicles related to ciliary transport or endocytotic vesicles. Preliminary data revealed endocytotic processes at the periciliary region of photoreceptor cells. In conclusion, we have identified Magi2 as component of the USH protein interactome. Present data indicate a role of SANS-Magi2 complex in endocytosis in the periciliary region of photoreceptor cells. These findings confirm the recently emphasized endocytotic function of the ciliary pocket in primary cilia. Furthermore, we provide first evidence for a molecular link of the USH protein interactome to the endocytosis machinery in photoreceptor cells.

